# Testing an Emerging Paradigm in Migration Ecology Shows Surprising Differences in Efficiency between Flight Modes

**DOI:** 10.1371/journal.pone.0035548

**Published:** 2012-04-25

**Authors:** Adam E. Duerr, Tricia A. Miller, Michael Lanzone, Dave Brandes, Jeff Cooper, Kieran O'Malley, Charles Maisonneuve, Junior Tremblay, Todd Katzner

**Affiliations:** 1 Division of Forestry and Natural Resources, West Virginia University, Morgantown, West Virginia, United States of America; 2 Riparia, The Pennsylvania State University, University Park, Pennsylvania, United States of America; 3 Cellular Tracking Technologies LLC, Somerset, Pennsylvania, United States of America; 4 Department of Civil and Environmental Engineering, Acopian Engineering Center, Lafayette College, Easton, Pennsylvania, United States of America; 5 Virginia Department of Game and Inland Fisheries, Fredericksburg, Virginia, United States of America; 6 West Virginia Division of Natural Resources, Romney, West Virginia, United States of America; 7 Ministère des Ressources naturelles et de la Faune, Rimouski, Québec, Canada; 8 Ministère des Ressources naturelles et de la Faune, Québec City, Québec, Canada; 9 United States Department of Agriculture, Forest Service, Timber and Watershed Laboratory, Parsons, West Virginia, United States of America; Institut Pluridisciplinaire Hubert Curien, France

## Abstract

To maximize fitness, flying animals should maximize flight speed while minimizing energetic expenditure. Soaring speeds of large-bodied birds are determined by flight routes and tradeoffs between minimizing time and energetic costs. Large raptors migrating in eastern North America predominantly glide between thermals that provide lift or soar along slopes or ridgelines using orographic lift (slope soaring). It is usually assumed that slope soaring is faster than thermal gliding because forward progress is constant compared to interrupted progress when birds pause to regain altitude in thermals. We tested this slope-soaring hypothesis using high-frequency GPS-GSM telemetry devices to track golden eagles during northbound migration. In contrast to expectations, flight speed was slower when slope soaring and eagles also were diverted from their migratory path, incurring possible energetic costs and reducing speed of progress towards a migratory endpoint. When gliding between thermals, eagles stayed on track and fast gliding speeds compensated for lack of progress during thermal soaring. When thermals were not available, eagles minimized migration time, not energy, by choosing energetically expensive slope soaring instead of waiting for thermals to develop. Sites suited to slope soaring include ridges preferred for wind-energy generation, thus avian risk of collision with wind turbines is associated with evolutionary trade-offs required to maximize fitness of time-minimizing migratory raptors.

## Introduction

Movement has dramatic consequences for demography and thus fitness [1]. Animals that undertake long-distance movements face trade-offs between minimizing time and minimizing energetic expenditures [2], [3]. Choosing incorrectly in these movements can have dramatic selective consequences [4], [5], [6].

Migration by birds progresses primarily through combinations of two flight types: straight-winged flight modes (soaring or gliding) and flapping flight. Knowing absolute and relative speeds of different flight types and modes is important to understand how energetically-or time-constrained animals move. Understanding flight speeds is also crucial to evaluating the influence of flight modes on the evolution of migration routes and wing morphology, and the complex trade-offs between time and energy when migrating [2], [3]. However, in spite of the importance of evaluating these processes, most studies that measure instantaneous or average flight speeds do not distinguish between different modes of flight [7], [8], [9], [10]. This is likely because comparison of speeds of different modes of soaring has been technologically difficult or impossible to achieve, even for large birds (e.g., [11], [12]).

**Figure 1:**
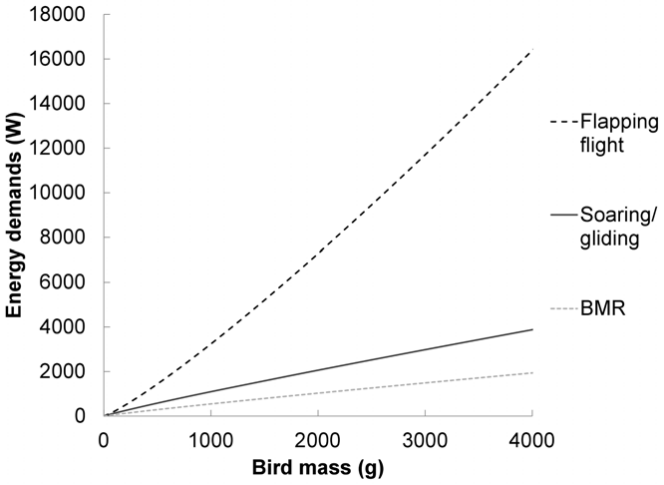
Energy demands of flight. Energy demands of flapping flight increase with body mass (E = M^1.17^; [13]) more rapidly than do basal metabolic rate (BMR = M^0.78^; [16]) or energy demands of soaring/gliding (2× BMR; [14], [15]). Schematic shows these relationships, assuming constant body and wing shape, size and wing loading.

Flight strategies used by large birds differ from those of small birds due to relationships between energetic costs of flight and bird mass. Although body shape, wing shape, and wing loading also affect flight energetics [11], energetic demands of flapping flight generally increase geometrically with body mass (E∝M^1.17^; [13]; Fig. 1). Thus, for birds with high body mass, energetic costs during flapping flight can be several times their basal metabolic rate (BMR). In contrast, energy required for soaring and gliding are proportionally lower, around twice that of BMR [14], [15]. Furthermore, BMR increases with body mass (BMR∝M^0.78^; [16]) at a much slower rate than energetic requirements of flapping flight; therefore, soaring becomes an increasingly efficient mode of flight as mass increases [17], [18].

Field observations support flight theory. Heavier species of harrier (*Circus* spp.) soared more and used flapping flight less than lighter harriers during migration [19]. The costs of flapping flight are dramatically apparent in observations of short-toed eagles (*Circaetus* gallicus) that extend migration routes 500–1700 km to avoid flapping flight over water [20] and griffon vultures (*Gyps fulvus*), among the heaviest flighted birds, which died at narrow sea crossings when forced only to use flapping flight [6].

Soaring is the use of air currents to aid in straight-winged flight with the two most prevalent modes over land being thermal and slope soaring [17]. First, thermal soaring is use of heated rising air to gain altitude. Differential heating of the earth causes surface layers of air to warm and rise, forming updrafts that can extend into lower layers of the atmosphere. Thermals develop during relatively calm conditions but break down with strong winds [21], [22]. Birds gain altitude by circling in these rising air currents during thermal soaring and they glide between them to make forward progress during migration [21]. For a given bird, flight speed during this glide depends on the glide angle, with steeper angles producing faster travel speeds but more rapid loss of altitude and potential energy [11]. Optimum glide angles depend upon a combination of the strength and spatial distribution of thermal updrafts [11]. Birds can make continuous forward progress and maintain altitude by gliding through thermals that are spatially aligned so that circling within thermals is not required. Such use of so called thermal streets (or straight-line gliding) should be a faster mode of flight than gliding between thermals because time is not lost circling in updrafts [11], [23].

Second, slope soaring depends upon horizontal winds and occurs when air is deflected upward by ridges, hills, or other structures. Such orographic lift develops only when winds are relatively fast and can be consistent and strong along a ridgeline allowing gliding in a manner similar to use of thermal streets [21], [29]. As with gliding between thermals, flight speed during slope soaring is dependent upon the strength of lift produced [21].

One often implicit (and occasionally explicit; [24]) hypothesis regarding flight speed is that slope soaring offers opportunity for greater migration speed than does thermal soaring and gliding. We refer to this as the slope-soaring hypothesis, formulated as follows. Soaring and gliding speeds are related to speed of vertical lift [11]. If lift is similar among updraft types then slope soaring should be faster than thermal soaring and gliding because wind provides a constant (uninterrupted) source of lift over ridges. In contrast, thermal flight requires interruption of forward progress to gain altitude while circling within a thermal. Although speed of gliding between thermals is faster than slope soaring [21], [25], falsification of this hypothesis requires that gliding speeds throughout migration must be fast enough to compensate for time spent soaring in thermals.

Weather influences development of thermal and orographic lift and thus use of respective types of soaring. There are also complex interactions among wind direction, wind speed, flight direction and flight speed [21], [23] with soaring birds responding to tail, head, and side winds by drifting with wind, compensating for wind, or both when choosing flight behavior [17], [26]. Therefore, weather influences on flight speed are dependent upon, and not separable from, choice of flight mode. Thus, although weather and flight interact strongly, testing the slope-soaring hypothesis does not require accounting for weather.

In the central corridor of the Appalachian Mountains of North America, different lift types have similar strengths, facilitating hypothesis testing. Along Pennsylvania's Kittatinny Ridge, for example, thermal lift of 1–4 m s^−1^ develops at discrete locations while cross winds produce vertical lift speeds up to 3–4 m s^−1^, providing soaring opportunities for a broad suite of raptors [21]. In this region, consistent cross winds also allow for development of wind-energy facilities along ridges used by migrating raptors [27]. For example, eastern golden eagles (*Aquila chrysaetos*) migrate through this corridor [28]. This population of eagles is small, likely numbering less than 2000 individuals, and faces increasing risks of collision with wind turbines with increasing development of wind-energy facilities in the Appalachian region [28]. Therefore, the potential for conservation conflict may be related to eagle's choices of flight modes.

We tested the slope-soaring hypothesis by tracking golden eagles with high-frequency GPS-GSM (global system for mobile communications) transmitters as the birds migrated through the central Appalachian Mountains. Golden eagles are an ideal species to test the soaring hypothesis because they are known to use both thermal and orographic lift during migration. The Ridge and Valley Province of the central Appalachian region facilitates such a test because opportunities for thermal and slope soaring are plentiful and ridges do not diverge greatly from the general axis of migration [21]. Support for the slope-soaring hypothesis must show that flight speed during slope soaring behavior is, on average, faster than flight speed during the combined phases of thermal soaring and gliding between thermals. However, the spatial distribution of thermal or orographic lift may not be perfectly aligned with the migratory path. Therefore, to incur evolutionary benefit, progress speed along an idealized-straight path must also be greater during slope soaring than combined thermal soaring and gliding. In our test, we compared both ground and progress speeds of golden eagles during thermal soaring, gliding between thermals, and slope soaring. To better understand the context for differences in flight speeds, we also tested for effects of slope of underlying terrain and altitude above ground level (AGL) on flight speeds.

## Materials and Methods

Eastern golden eagles breed in Québec, Labrador and Ontario, Canada, and migrate south through the northern and central Appalachian Mountains, from Maine to Virginia, USA [28].

**Figure 2:**
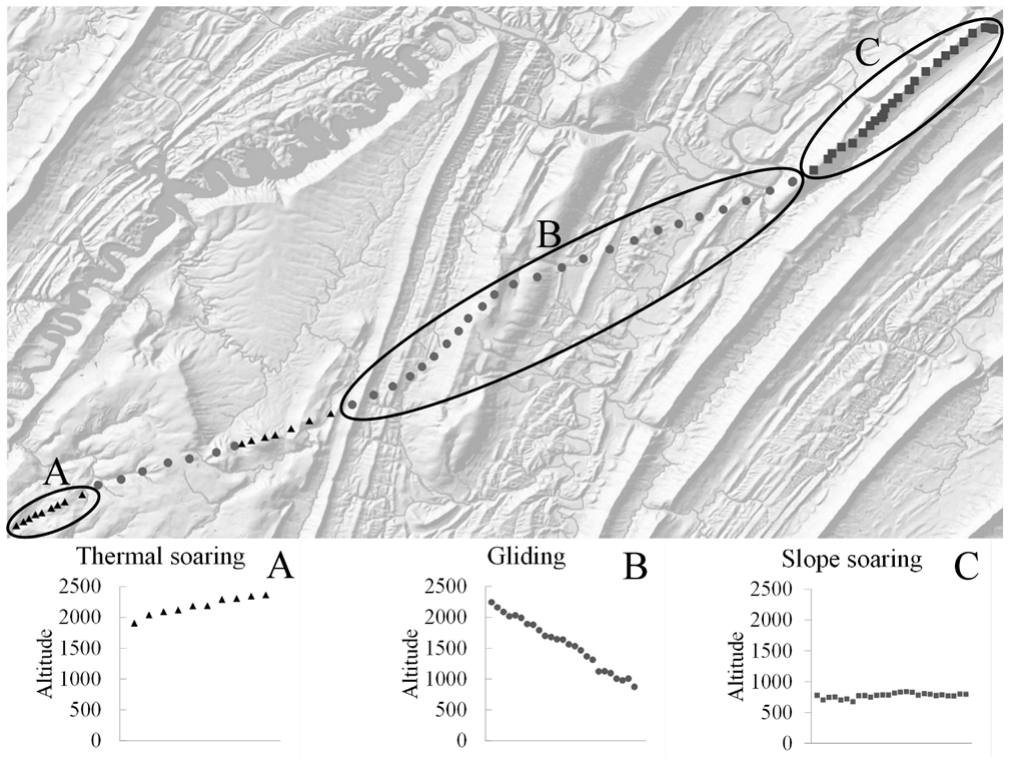
Altitude and topography of flight types. Flight modes were manually classified based upon flight patterns, changes in flight altitude, and underlying topography for locations of golden eagles during spring migration 2009–2010. A) Thermal soaring includes points (triangles) that are closely spaced with increasing altitude. B) Gliding points (circles) connect flight segments of thermal soaring, with points decreasing in altitude. C) Slope soaring segments include locations (squares) along ridgelines that are close to ground level (less than 200 m). The background map shows topographic relief with darkened slopes of ridgelines.

We captured five (three subadult and two adult male) golden eagles during winters 2009–2010 in Pennsylvania, Virginia and West Virginia, USA, using cannon or rocket nets baited with roadkill-deer carcasses. Eagles were outfitted with CTT-1100 GPS-GSM telemetry systems (Cellular Tracking Technologies, LLC) attached as backpacks with Teflon ribbon [29] and released. CTT-1100 s collect and save GPS data and transmit them through the GSM network. We programmed transmitters to collect data at 30 s intervals while the bird was flying between latitudes 39.5° and 42.5° north during spring migration. Data were post-processed and manually classified into flight modes by a single observer (TAM). Flight modes were identified based upon patterns of sequential GPS locations (Fig. 2). Closely spaced points in which an eagle gained altitude were characterized as thermal soaring. Points between thermals in which eagles lost altitude were characterized as gliding. Slope soaring was inferred from points that followed ridgelines and that stayed within a narrow altitudinal band (observed max AGL = 450 m). Our unit of analysis was the flight segment, which we defined as discrete series of GPS points that were the same flight mode and that were separated by less than 90 s.

**Figure 3:**
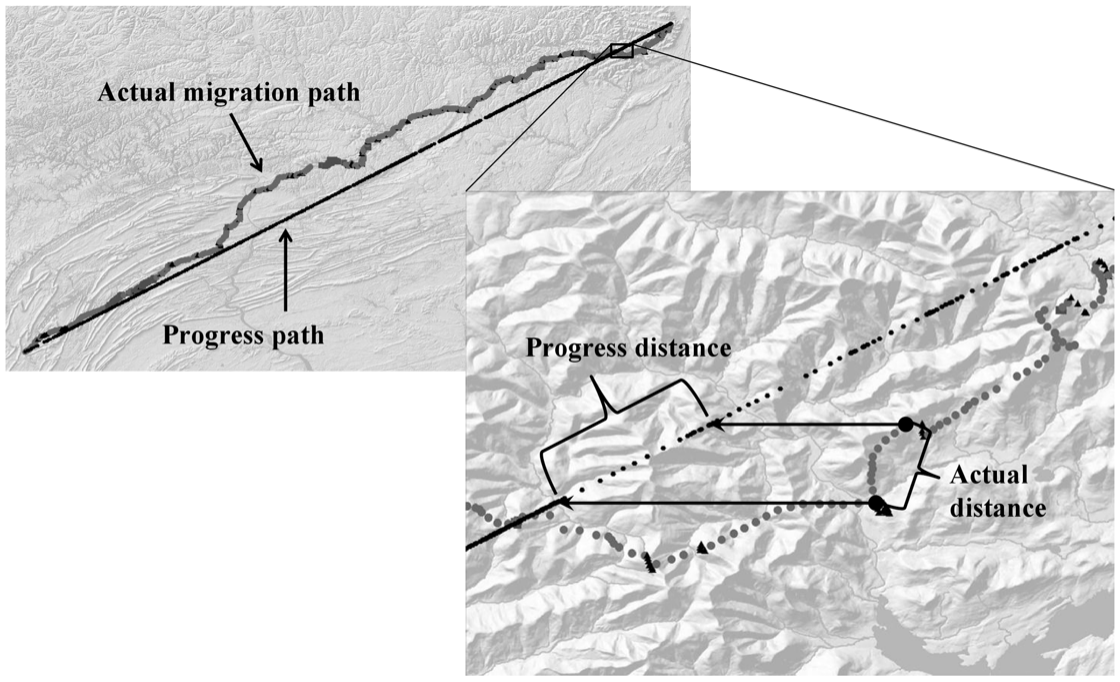
Progress path and progress distance. The progress path is the straight line that connects the first and last points that a satellite tag recorded for each golden eagle as it migrated from the 39.5° to the 42.5° north latitude. For each flight segment, the progress distance is the distance along the progress path defined by the latitudes of the start and end points of the segment. The progress speed is the quotient of the progress distance for the segment and the time an eagle spent traveling along the actual path of the segment.

For each flight segment, we calculated two flight speeds. First, ground speed, the speed at which an eagle moved relative to the ground, was calculated as the average of instantaneous flight speeds recorded by the telemetry device within a discrete flight segment. Second, progress speed is flight speed relative to a functional distance (the progress path) traveled during migration [30]. One likely measure of the functional distance is the idealized direct path through latitudes 39.5° and 42.5° north. Thus, we defined the progress path as the straight-line from an eagles' first location to its last location at these latitudes (Fig. 3). We then identified the start and end points for each flight segment by drawing horizontal lines from the start and end of the actual segment traveled to intersect points along the idealized progress path. The progress speed for a segment was calculated as the distance between the start and end points on the progress path divided by the amount of time the eagle traveled along the flight segment.

We used a step-wise procedure to build linear mixed models (PROC MIXED, SAS v. 9.2) for flight speed. We used flight mode, slope, and AGL as explanatory variables to build separate models for ground and progress speed. We only used flight modes of gliding and slope-soaring in models because only two levels are required to differentiate three categorical factors. We used *p*<0.05 for a factor to enter the model and *p*<0.10 for a factor to remain in the model at each step.

## Results

We identified 578 distinct flight segments from five golden eagles. The number of flight segments per eagle ranged from 10 to 78 for gliding (n = 276 segments total; 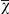 = 55.2±12.06 (±se)), 14 to 71 for thermal soaring (n = 261; 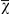 = 52.2±11.37), and 1 to 17 for slope soaring (n = 41; 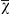 = 8.2±2.56). The average recorded duration of a cycle of thermal soaring and gliding was longer (340 s) than flight segments of slope soaring (220 s). During spring migration, eagles used thermal soaring and gliding nine times more than slope soaring.

Flight speed differed dramatically by flight mode and with AGL (Table 1, 2; Table S1). Ground and progress speeds of gliding between thermals was fastest, slope soaring was intermediate, and thermal soaring was slowest (Table 1). Empirical estimates of average ground speeds closely matched most ground speeds predicted by the linear model (differences ≤0.7 m s^−1^; Table 1). Raw progress speeds for thermal soaring and soaring and gliding were slightly different (by 2.2 to 3.0 m s^−1^) than those predicted by our model, although relative relationships among flight modes were identical in all cases. Ground speed in a glide was 67% faster than when slope soaring, and thermal soaring and gliding combined was 32% faster than slope soaring. Differences in progress speeds between flight modes were more pronounced than differences between ground speeds. Progress speed for gliding was 138% faster than slope soaring, and thermal soaring combined with gliding was 31% faster than slope soaring.

**Table 1:** Mean ± s.e. ground and progress speeds for flight modes used by five golden eagles migrating through Pennsylvania during spring migration, 2009–2010.

Speed		Mean raw speed (m s^−1^)	Mean modeled speed (m s^−1^)
Ground	Thermal soaring	10.45±0.80	10.26±0.82
	Slope soaring	10.90±0.87	11.49±0.95
	Thermal soaring & gliding	14.24±0.78	14.48±0.82
	Gliding	18.07±1.39	18.04±0.82
Progress	Thermal soaring	1.87±1.54	4.73±0.74
	Slope soaring	7.35±0.96	7.79±1.01
	Thermal soaring & gliding	9.59±1.08	11.72±0.74
	Gliding	17.32±1.44	17.61±0.75

Raw speeds are the average of speeds measured for each bird (empirical estimates). Modeled speeds are predictions from linear mixed models (see table 1). *N* = 5 in all cases.

**Table 2:** Model parameters ± s.e. and statistics for effects that influence ground and progress speed (m s^−1^) of golden eagles as they passed through the central Appalachians during spring migration, 2009–2010.

Speed		Intercept*	Gliding	AGL†	Slope soaring
			(m s^−1^)	(m)	(m s^−1^)
Ground	Model coefficients	8.910±0.847	7.020±0.284	0.002±0.001	2.070±0.543
	*F*_1,368_	-	610.75	43.49	14.56
	*p*	-	<0.0001	<0.0001	0.0002
Progress	Model coefficients	2.244±0.796	11.471±0.433	0.005±0.001	4.605±0.816
	*F*_1,369_	-	701.51	70.26	31.86
	*p*	-	<0.0001	<0.0001	<0.0001

* Intercept term includes values for thermal soaring.

† Altitude above ground level.

Variance-covariance matrices for each model are given in Table S1.

Flight speed was influenced by AGL (Table 2). For every 1000 m increase in AGL, ground speed increased by 2 m s^−1^ and progress speed increased by 5 m s^−1^. As expected, AGL was greater, on average, for thermal soaring (541 m) and gliding (846 m) than slope soaring (204 m, Table 3). Slope of the underlying terrain did not influence ground (*F*_1,367_ = 2.78, *p* = 0.096) or progress (*F*_1,369_ = 0.05, *p* = 0.826) speeds and was not added to the final model.

**Table 3:** Means ± se of predictor variables and time measures for flight modes used by five golden eagles that migrated through Pennsylvania during spring, 2009–2010.

Flight mode	No. flight segments*	Altitude above ground level (m)	Slope (deg)	Time per segment (s)	Proportion of total time
Thermal soaring	261	541±76.5	11.4±1.05	158±29.4	0.411±0.045
Slope soaring	41	204±29.1	17.3±1.56	223±63.2	0.102±0.039
Gliding	276	846±118	10.6±0.56	187±33.1	0.487±0.041

* For reference only, *n* = 5 eagles for all measures.

## Discussion

Evaluating the trade-off between minimizing time or energy depends on knowing how different movement choices benefit an organism. Flight speed of migrants determines how quickly temporally or energetically constrained individuals reach critical breeding or wintering areas. Therefore, the consequences of the flight mode that birds choose have important selective relevance to evaluate and interpret this trade-off.

The slope-soaring hypothesis reflects prevailing thought in flight theory; however, it is not supported by this analysis. Golden eagles that soar in and glide between thermals flew faster than when slope soaring. This was true for both ground and progress speeds and ran contrary to the predictions of the hypothesis we tested. Ground speeds were faster in thermal-powered flight because the extremely high flight speed during gliding more than compensated for interruptions caused by thermal soaring to gain altitude.

In terms of making progress toward a migratory goal, gliding between thermals was dramatically faster than slope soaring. The reasons for this became more obvious when comparing ground and progress speeds. During thermal soaring, progress speed was much slower than ground speed because forward progress is offset by backward progress as birds circle. When gliding between thermals, ground and progress speeds were nearly equal, meaning eagles made rapid forward progress along their preferred migratory pathway (this also served as validation of our choice of an idealized path). However, when slope soaring, progress speed was 33% slower than ground speed. This was because eagles were diverted from their preferred migratory pathway when following ridgelines to take advantage of orographic lift. Thus, although slope soaring follows a more constant lift source, it constrains the birds to a specific topographic feature and is, therefore, less efficient than gliding between thermals, a behavior in which the eagle can largely choose its flight direction.

Although birds that soar use multiple flight modes, they are rarely presented with the opportunity to choose between modes when migrating. Thermals develop during calm conditions while orographic lift is available when wind speeds are fast [21], [22]. Therefore, the potential trade-off that exists on any given day is rarely a choice between thermal- and orographically-powered flight, but instead a choice between not flying or using a less energetically efficient mode of flight (orographic lift) to reach a migratory endpoint (breeding or wintering sites). This logic suggests that birds using orographic lift are more likely to be evolutionarily constrained by time, for example, a desire to reach breeding grounds as early as possible, rather than a desire to conserve energy by waiting for ideal flight conditions (thermals).

Soaring birds trying to minimize energy expenditures should pause on migration when the energetic costs of suboptimal flight are greater than energy spent not migrating. Energy expenditures during soaring and gliding are two times BMR [14], [15]; therefore, birds trying to minimize energetic costs should pause during migration whenever progress speed of slope soaring is approximately 50% of progress speed for thermal soaring and gliding (10.4 m s^−1^). This prediction assumes that eagles only rest when pausing; however, golden eagles forage while on migration (authors, unpublished data). When birds use a “fly and forage” migration strategy, energy gained during pauses in migration provide resources used during future migratory flights [31]. Therefore, an energy-minimization migration strategy would call for pausing to forage during migration instead of following ridgelines that would divert a bird from its preferred migratory pathway.

Soaring birds trying to minimize time spent on migration may be forced to choose forward progress over energy conservation during part of migration. If golden eagles minimize energy expended during migration, they should not use orographic lift when progress speeds during slope soaring drop below half of the progress speed of thermal soaring and gliding. However, nearly half (46%) of the slope-soaring speeds we recorded were slower than this threshold (5.2 m s^−1^), suggesting that much of the time, different considerations drive migratory decisions of eagles. It appears instead that the golden eagles we monitored minimized migration time by choosing to slope soar at the expense of expending energy during spring migration. Indeed, there is selective pressure on many migratory species to arrive early on breeding grounds to occupy limited high-quality breeding sites, to commence breeding during short breeding seasons, and to maximize productivity [4], [5]. If this explanation of the rationale for time vs. energy minimization strategies by eagles is correct, we would predict that immature eagles, which face no pressure to arrive early on breeding grounds, would be energy, instead of time, minimizers. Such birds should therefore wait until later in the season when thermals predominate [22], flight is more direct, and thus energetic costs of migration should be lower. Preliminary observations support this prediction (the authors unpublished data).

Other factors may also influence flight modes and flight speed. Of these, weather conditions are among the most important (although testing our hypothesis does not require an understand of weather). For example, wind direction influences flight speeds and flight energetics; ground speed should increase with tailwinds and decrease with head and side winds [21], [23]. Also, sources of lift change seasonally, with orographic lift predominating during fall [22]. Our data are likely representative of all wind conditions experienced by eagles for each flight mode but are limited with regard to seasonality, suggesting future avenues for continued exploration of the flight speed problem. Finally, certain flight modes may provide hunting opportunities during active migration. Eagles may encounter prey frequently when slope soaring at low altitudes, or may maximize search efficiency when thermal soaring over one area. As noted earlier, flight mode, flight speed, and lift impact demography because of their evolutionary relevance. There is also applied relevance to understanding flight speed because the trade-off between these evolutionary choices also interact to influence risk of eagles colliding with wind turbines. During windy conditions, orographic lift develops along steep terrain and extends upward to flatter ridgelines. Eagles use this resource to subsidize migration, flying at moderate speeds. Orographic lift begins to degrade quickly (at about 200 m AGL in normal wind) at the top of ridges [21]; therefore, birds are restricted to relatively low flight altitudes, which put them in the rotor-swept zone of modern horizontal-axis turbines. As winds increase, orographic lift increases, and birds may fly higher and faster over ridges and avoid turbines [32]. When winds are calm, thermals develop and eagles soar using thermal lift and gliding. As thermal lift increases, flight speed and flight AGL increase. As in the case of slope soaring, risk of collision with wind turbines is lower at faster flight speeds because as flight altitude increases, risk decreases.

For soaring birds, risk of collision with wind turbines is therefore dependent upon flight mode, but moderated by flight speed. Because annual survival for some species is predominantly determined by survival on migration [33], and because collision with turbine blades is an important source of mortality for some golden eagles [34], consequences of flight mode and flight speed may be an important determinant of demographic impacts from wind development to golden eagle populations.

By refuting the slope soaring hypothesis, this work allows us to better understand the evolutionary tradeoffs underpinning flight behavior. We also highlight an emerging conflict between soaring flight and increased risk of mortality from wind-energy development, while making specific predictions about the relationship between that risk and flight mode.

## Supporting Information

Table S1 **Variance-covariance matrix for linear models (** **Table 2** **) that describe flight speed of golden eagles as they passed through the central Appalachians during spring migration, 2009–2010.**
(DOC)
