# Supplementary material for: Testing an Emerging Paradigm in Migration Ecology Shows Surprising Differences in Efficiency between Flight Modes
Source: PLoS One. 2012 Apr 25;7(4):e35548. doi: 10.1371/journal.pone.0035548 (PMC3338847; doi:10.1371/journal.pone.0035548)
Supplement: Table S1 — Variance-covariance matrix for linear models ( Table 2 ) that describe flight speed of golden eagles as they passed through the central Appalachians during spring migration, 2009–2010. (DOC) [file pone.0035548.s001.doc]

|  |  | Intercept* | Glide (m s^-1^) | AGL† (m) | Slope soar ‡ (m s^-1^) |
| --- | --- | --- | --- | --- | --- |
| Ground | Intercept | 0.71760 |  |  |  |
| Speed | Glide | -0.00871 | 0.08068 |  |  |
|  | AGL | -0.00008 | -0.00005 | 0.00000 |  |
|  | Slope soar | -0.04291 | 0.02589 | 0.00003 | 0.29440 |
|  |  |  |  |  |  |
| Progress | Intercept | 0.63430 |  |  |  |
| Speed | Glide | -0.02090 | 0.18760 |  |  |
|  | AGL | -0.00016 | -0.00010 | 0.00000 |  |
|  | Slope soar | -0.12870 | 0.05908 | 0.00007 | 0.66560 |

^*^Intercept provides variance-covariance values for thermal soaring.

†Altitude above ground level

‡Slope soaring
